# Argovit™ Silver Nanoparticles Effects on *Allium cepa*: Plant Growth Promotion without Cyto Genotoxic Damage

**DOI:** 10.3390/nano10071386

**Published:** 2020-07-16

**Authors:** Francisco Casillas-Figueroa, María Evarista Arellano-García, Claudia Leyva-Aguilera, Balam Ruíz-Ruíz, Roberto Luna Vázquez-Gómez, Patricia Radilla-Chávez, Rocío Alejandra Chávez-Santoscoy, Alexey Pestryakov, Yanis Toledano-Magaña, Juan Carlos García-Ramos, Nina Bogdanchikova

**Affiliations:** 1 Escuela de Ciencias de la Salud, UABC, Blvd. Zertuche y Blvd., De los Lagos S/N Fracc, Valle Dorado, 22890 Ensenada, Baja California, Mexico; casillas.francisco@uabc.edu.mx (F.C.-F.); cuentarluna@gmail.com (R.L.V.-G.); patyradilla@uabc.edu.mx (P.R.-C.); yanis.toledano@uabc.edu.mx (Y.T.-M.); 2 Facultad de Ciencias, UABC, Carretera Transpeninsular Ensenada-Tijuana No. 3917 Col. Playitas, 22860 Ensenada, Baja California, Mexico; cleyva@uabc.edu.mx; 3 Facultad de Medicina extensión los Mochis, Universidad Autónoma de Sinaloa, Av. Ángel Flores s/n, Ciudad Universitaria, 81223 Los Mochis, Sinaloa, Mexico; balamruiz@gmail.com; 4 Centro de Biotecnología-FEMSA, Escuela de Ingeniería y Ciencias, ITESM, Monterrey, Eugenio Garza Sada, 2501 Sur, 64849 Monterrey, Nuevo León, Mexico; chavez.santoscoy@tec.mx; 5 Department of Technology of Organic Substances and Polymer Materials, Tomsk Polytechnic University, 634050 Tomsk, Russia; pestryakov2005@yandex.ru; 6 Centro de Nanociencias y Nanotecnología, UNAM, Carretera Tijuana-Ensenada Km 107, 22860 Ensenada, Baja California, Mexico; nina@cnyn.unam.mx

**Keywords:** nongenotoxic silver nanoparticles, genotoxic, cytotoxic, antioxidant activity, silver ions, *Allium cepa*, metal/coating agent ratio

## Abstract

Due to their antibacterial and antiviral effects, silver nanoparticles (AgNP) are one of the most widely used nanomaterials worldwide in various industries, e.g., in textiles, cosmetics and biomedical-related products. Unfortunately, the lack of complete physicochemical characterization and the variety of models used to evaluate its cytotoxic/genotoxic effect make comparison and decision-making regarding their safe use difficult. In this work, we present a systematic study of the cytotoxic and genotoxic activity of the commercially available AgNPs formulation Argovit™ in *Allium cepa*. The evaluated concentration range, 5–100 µg/mL of metallic silver content (85–1666 µg/mL of complete formulation), is 10–17 times higher than the used for other previously reported polyvinylpyrrolidone (PVP)-AgNP formulations and showed no cytotoxic or genotoxic damage in *Allium cepa*. Conversely, low concentrations (5 and 10 µg/mL) promote growth without damage to roots or bulbs. Until this work, all the formulations of PVP-AgNP evaluated in *Allium cepa* regardless of their size, concentration, or the exposure time had shown phytotoxicity. The biological response observed in *Allium cepa* exposed to Argovit™ is caused by nanoparticles and not by silver ions. The metal/coating agent ratio plays a fundamental role in this response and must be considered within the key physicochemical parameters for the design and manufacture of safer nanomaterials.

## 1. Introduction

Silver nanoparticles (AgNPs) are the most widely used nanomaterials worldwide in different areas such as the pharmaceutical, food, biomedical, textile and agricultural industries, due to their high capacity as antimicrobial and antiviral agents [1,2,3]. Due to the AgNPs diverse areas of application, it is fundamental to know, as much as possible, the toxicological profile of each nanoparticle formulation. 

Physicochemical properties of AgNPs, such as size, shape, stability, and the coating agents have been identified as direct modulators of the cytotoxic/genotoxic damage elicited on different cellular systems, e.g., mammals, plants, bacteria [4,5,6,7,8,9]. Practically all publications identify the release of silver ions and reactive oxygen species (ROS) overproduction as triggers of cellular damage. Additionally, many of them described size-dependent toxicity, as the smaller the nanoparticles, the higher the toxicity found [1,4,9,10,11,12,13].

Conversely, several works associate the cytotoxic damage not to the released silver ions but to the nanoparticle itself [14,15,16,17,18]. Furthermore, it was found that the coating agent could play a significant role in the cytotoxic/genotoxic damage and the cellular uptake by a dependent or independent clathrin/caveolae endocytosis [19,20,21,22,23].

Just a few AgNPs formulations provide a complete characterization, and even fewer have been evaluated on diverse systems, including those recognized as a reference, i.e., primary cultures in the case of mammals [24] and *Allium cepa* for higher plants [25]. The above mentioned makes the task of comparison and decision-making regarding toxicity and safety use of AgNPs very difficult. 

*Allium cepa* is considered one of the most sensitive plant systems to determine the cytotoxic and genotoxic effects of diverse chemical agents. The advantage provided by this system has been widely described in different works and reviews articles [25,26,27,28]. Despite all known benefits, the use of this model for nanomaterials still provides controversial results that made hard the task for decision-makers. Most of the problems are not associated with the model itself but to the scarce physicochemical properties of nanomaterials supplied by the authors. Furthermore, in our knowledge, scarce studies reported the physiological response of plants exposed to different concentrations of silver ions and fewer with the coating agent alone. 

Diverse biological responses were described when *Allium cepa* was exposed to various formulations of AgNPs. The observed effects were mainly associated with the silver ions released. However, different groups working with very stable AgNPs formulations—most of them coated with polyvinylpyrrolidone (PVP)—showed cytotoxic and genotoxic effects that cannot be associated with the leached silver ions [14,15]. Thus, the biological response must be elicited mainly by the nanoparticle itself and not by its constituents. 

Table 1 summarizes the cytotoxic and genotoxic response registered after the exposure of *Allium cepa* to different concentrations of diverse AgNPs formulations from published data. Most of the formulations assessed reported cytotoxic and genotoxic damage, mainly those that lack of coating agent and the biogenically produced nanoparticles [29,30,31,32,33,34,35,36,37]. 

It is well known that the coating agent contributes to the stability of AgNPs and, in turn, their toxicity response [3,21,22,38,39]. The use of PVP as a coating agent substantially diminishes the genotoxic damage [40,41]. Minimal effect on root elongation and the mitotic index were found with AgNPs coated with citrate (61.2 nm), PVP (9.4 nm) and CTAB (5.6 nm) [14]. Interestingly, all AgNPs evaluated by Cvjetko [14] produce cytotoxic damage, increasing ROS concentration, and lipoperoxidation with an AgNPs concentration-dependence manner. Although no DNA damage was observed with citrate-AgNPs by comet assay (Table 1).

An essential contribution of the manuscript of Cvjetko is the association of cytotoxic and genotoxic damage to the nanoparticles and not to the released silver ions [14]. Another work by Scherer [15] also reported the cytotoxic and genotoxic effect of PVP-AgNPs with different sizes with no contribution of free Ag^+^ ions to cytotoxicity observed. In the mentioned work, the authors describe a cytotoxic and genotoxic effect with a size dependence behavior. Small nanoparticles produce more considerable cytotoxic damage and micronuclei (MN) frequency. All AgNPs studied in this work, no matter the size, produce cytotoxic and genotoxic damage. 

**Table 1:** Comparative analysis of cyto-genotoxicity of Argovit™ AgNPs with other AgNPs formulations described in the literature.

AgNP Source and Physicochemical Characteristics	Shape	Size (nm)	ζ ^a^ (mV)	Ag Content ^b^	RP ^c^ (nm)	[C] ^d^ (µg/mL)	Exposure Time (h)	Cytotoxic and Genotoxic Damage	Ref.
Commercial Sigma-Aldrich,	<100	-	-	99.5%	-	25, 50, 75 and 100 µg/mL	4 h	CA and cell disintegration.	[29]
Commercial Sigma-Aldrich	<100	-	-	99.5%	-	5, 10, 20, 40, 80 µg/mL	2 h of exposure and recovery of 12, 24 and 48 h	20 and 40 µg/mL. Dose-dependence increase in the frequency of cells with MN and CA ≥10 µg/mL: DNA damage (comet assay)	[37]
Synthesized with male inflorescence of screw pine, *Pandanus odorifer*		-	-	-	-	5, 10, 20, 40 and 80 µg/mL	2h of exposure and recovery of 12, 24 and 48 h	Dose-dependence increase in the frequency of cells with CA After 2 h of exposure and 48 h of recovery, no differences in cells with MN between control and lower concentrations (5 and 10 µg/mL). ≥20 µg/mL: DNA damage (comet assay)	[37]
Commercial Sigma-Aldrich	-	TEM 70–130, av. ~125; SEM: 90–180, av. 120	−4.86	99.5%	-	25, 50 and 75 µg/mL	24 h	No damage was observed in nuclei isolated from shoots. Nuclei isolated from roots exposed to 25 and 50 µg/mL shown DNA damage determined by comet assay. The major effect was observed with 50 µg/mL. No damage was observed with 75 µg/mL, and the authors suggest agglomeration and precipitation of AgNP.	[42]
Synthesis AgNP-citrate AgNP-PVP AgNP-CTAB	Citrate rod-like PVP spherical CTAB spherical	Citrate 61.2 ± 33.9 (TEM) PVP 9.4 ± 1.3 (TEM) CTAB 5.6 ± 2.1 (TEM)	Citrate −39.8 ± 3.4 PVP −4.8 ± 0.6 CTAB 42.5 ± 2.7	-	-	25, 50, 75, 100 µM (Quantified by ICP-MS) 10 µM AgNO_3_, 2.5, 5.0, 7.5, 10 µg/mL	72 h	No DNA damage was observed with any of the AgNP-citrate concentrations employed. An increase in tail DNA was recorded after exposure to AgNP-PVP at 100 μM concentration. AgNP-CTAB produces DNA damage only with 50 μM concentration.	[14]
Commercial Nanotech PVP-AgNPs	-	20–30	-	--		5, 10, 15 µg/mL	3, 6, 9 h	The decrease in MI and the increase in CA have a dependence on concentration and exposure time	[43]
AgNPs Synthetized with leaf extract of *Swertia chirata* Commercial Sigma-Aldrich	-	Synthesis 20 Commercial 20	-	-		5, 10, 20 µg/mL	4 h	The decrease in MI and the increase in CA have a dependence on concentration. Both AgNPs produce cytotoxic and genotoxic damage similar to AgNO_3_.	[30]
They were synthesized with *Cola nitida* pod (p), seed (s), and seed shell (ss).	All semi-spherical	p: 12–80 s: 8–50 ss: 5–40	-	-	p: 431 s: 457 ss: 454	0.01, 0.1, 1, 10 and 100 µg/mL	24, 48 and 72 h	Cytotoxic and genotoxic damage have a dependence on concentration and exposure time.	[31]
Synthesized with plant extract	Semi-spherical	25–40	-	-	440	1, 5 and 10 µg/mL	72 h	Produces a reduction in the number and diameter of roots, decreases in MI, and increases the frequency of CA.	[33]
Synthesized AgNPs	-	2–8	-	-	-	1.5 and 15 µg/mL With CMC 1.24 and 12.4 µg/mL	24 h	Cytotoxic and genotoxic effects with concentration-dependence behavior (MI decrease and CA increase). In the presence of CMC, the cytotoxic damage is lower than the observed for AgNPs alone. Genotoxic damage is found only with 12.4 µg/mL.	[34]
Synthesized with Althea officinalis leaf extract (E) and dehydrated root infusion (R)	-	E: 157 ± 11 (DLS), 131 ± 5 (NTA) R: 293 ± 12 (DLS) 227 ± 16 (NTA)	E: 20.1 ± 1 R: 26.0 ± 1	E: 7.2 × 10^10^ NP/mL (NTA); R: 4.6 × 10^10^ NP/mL	E 384 R 380	E: 3 × 10^10^ NP/mL (3.4 µg/mL) R: 3 × 10^8^ NP/mL	24 h	An increase in MI and CA observed. AgNPs produce a frequency increase on cells with chromosome damage more than 3-times compared with control, but the extract of *Althea officinalis* produces a frequency increase of nearly 3-times	[35]
Biogenic AgNPs obtained with *Fusarium oxysporum.* Unwashed (AgNPuw) and washed (AgNPw) with water	-	AgNPuw 40.3 ± 3.5 (TEM) 106.2 ± 13 (DLS) AgNPw 40.3 ± 3.5 (TEM) 145.1 ± 4.5 (DLS)	AgNPuw −37.1 ± 2.6 AgNPw −47.8 ± 1.1	-	-	0.5, 1, 5 and 10 µg/mL	24 h	No difference in the MI compared with control, but 5 and 10 µg/mL of AgNPs increase the frequency of CA. No data of lower concentration was provided. Results of genotoxicity at concentrations 5.0 and 10.0 ug/mL show some response, but at concentrations 0.5 and 1.0 μg/mL, the washed and unwashed silver nanoparticles did not present any effect.	[36]
Commercial BioPure Silver Nanospheres–PVP (5, 25, 50, 75 nm) PVP: 40 kDa from nanoComposix^®^ Characterization performed by the authors BE: before exposure AE: After exposure	All nanoparticles are spherical	AgNP5 size: 10.4 ± 4.7 nm (TEM). BE d: 42.6 ± 19.2 nm (DLS); AE d: 161.2 ± 55.5 nm (DLS) AgNP25 size: 20.4 ± 7.2 nm (TEM) BE d: 77.1 ± 26.2 nm (DLS); AE d: 94.5 ± 42.9 nm (DLS) AgNP50 size: 51.3 ± 7.4 nm BE d: 80.5 ± 30.4 nm (DLS); AE d: 103.3 ± 46.5 nm (DLS) AgNP75 size: 73.4 ± 4.7 nm BE d: 124.4 ± 48.1 nm (DLS); AE d: 119.8 ± 42.1 nm (DLS)	AgNP5 BE −15.6 AE −8.35 AgNP25 BE −11.2 AE −6.81 AgNP50 BE −16.3 AE −7.53 AgNP75 BE −13.0 AE −6.42	AgNP5 Release of Ag^+^ from PVP-AgNPs in distilled water: 0.75% AgNP25 Release of Ag^+^ from PVP-AgNPs in distilled water: 0.29% AgNP50 Release of Ag^+^ from PVP-AgNPs in distilled water: 0.03% AgNP75 Release of Ag^+^ from PVP-AgNPs in distilled water: < LOQ	-	100 µg/mL	48 h	The smaller the AgNPs diameter, the more the MI decrease, the MN frequency increases compared to the control group	[15]
Synthesized AgNPs with cocoa pod husk (A = CPHE-AgNPs) and cocoa bean (B = CBE-AgNPs)	A 4–32 (TEM) B 8.9–54.2 (TEM)	-	-	-	A 428 B 438	0.01, 0.1, 1, 10 and 100 µg/mL	24, 48 and 72 h	Cytotoxicity and genotoxicity shown dependence on concentration and time exposure	[32]

^a^: Zeta potential; ^b^ resonance plasmon; ^c^ content of silver in the AgNPs formulation; ^d^ concentration used in the experiments; MI: mitotic index; CA: chromatic aberrations; PVP: polyvinylpyrrolidone; CTAB: cetyltrimethylammonium bromide; CMC: carboxymethylcellulose; LOQ: limit of quantification; BE: before exposure; AE: after exposure.

During the last years, our research group has studied a commercial PVP-AgNPs formulation known as Argovit™ that has shown striking results in agriculture, aquaculture, and human and veterinary medicine [44,45,46,47,48,49,50,51]. These AgNPs have been very useful in disinfection and heal acceleration of diabetic wounds [44], reduction of tumor growth on mice [45], treatment of white spot virus on shrimps without toxic effects [46,47,48,49,50,51,52] and distemper on dogs [47], a decrease of the infectivity of Rift Valley fever virus on mice [48], elimination of parasites from fish for human consumption [49], disinfection and promotion of plants growth during micropropagation [50,51], among many others.

In this work, we present the systematic study of *Allium cepa* biological response elicited by the exposure for 24, 48 and 72 h to different concentrations of a fully characterized PVP-AgNPs formulation, silver ions from AgNO_3_ solution corresponding to the amount of silver contained in the nanoparticles and the corresponding amount of PVP (acting as coating agent of the nanoparticles) for each concentration assessed. The physiological response was evaluated, monitoring the number and length of new roots. The cytotoxic damage was determined considering the mitotic index, the effects on the mitosis cycle, and the evaluation of ROS overproduction, the antioxidant response of the onion, quantification of the total phenol content, and evidence of lipoperoxidation. Finally, the endpoint to determine genotoxic damage was the change in the micronuclei frequency on dividing cells.

## 2. Materials and Methods

### 2.1. Materials

The AgNPs formulation used in this work is a stable aqueous suspension that contains 1.2% weight of metallic silver stabilized with 18.8% weight of PVP, commercially available as Argovit™. The final concentration of the suspension is 200 mg/mL (20%) of AgNPs. The AgNPs of this formulation has been described as a spheroidal shape by transmission electron microscopy (TEM) with a diameter distribution between 1 to 90 nm and an average size of 35 ± 12 nm. The hydrodynamic diameter is 70 nm, with a zeta potential of −5 mV and a plasmon resonance found at 420 nm. All determinations performed in distilled water [45]. Silver nanoparticles were donated by Vasily Burmistrov of Vector-Vita Scientific and Production Center (Novosibirsk, Russia). The UV-vis, zeta potential, and hydrodynamic diameter for AgNPs batch used in this work were determined in distilled water. The UV-vis spectra were acquired with an Agilent Cary 60 spectrophotometer (Agilent Technologies, Santa Clara, CA, USA), and the absorption maximum was observed on 424 nm. The zeta potential (−14 mV) and the hydrodynamic diameter (95 nm) were determined with a Zetasizer Nano NS DTS-1060 (Malvern Panalytical Ltd., Worcestershire, UK) The values obtained agree with those reported by the producers.

### 2.2. Experimental Design

For each treatment, three *Allium cepa* bulbs (2–3 cm of diameter) were used. Roots were removed without primordial destruction. After washing, each bulb was placed in a 50 mL Falcon conical tube. Each tube contained 10 mL of distilled water and the corresponding treatment: AgNPs, AgNO_3_, or PVP. The final concentrations for AgNPs and AgNO_3_ were 5, 10, 15, 25, 50, 75 and 100 µg/mL (metallic silver content), while the final PVP concentrations were 78, 156, 235, 391, 783, 1175 and 1566 µg/mL. The PVP concentrations correspond to the maximum amount of polymer used as a coating agent on each AgNPs concentration evaluated, considering that Ag%: PVP% ratio in Argovit™ is 1.2%:18.8%. Distilled water was used as a negative control (C−) and sodium arsenite (NaAsO_2_) at a concentration of 0.37 µg/mL (2.84 µM) as a positive control (C+). The inclusion of positive genotoxic control is to guarantee that the cyto-genotoxic response observed is a product of the agents studied and not an artifact of the technique. Samples were incubated at 25 °C ± 0.5 °C for 72 h in darkness with the corresponding stimuli, except NaAsO_2_ samples, which were exposed only for one hour with the stimuli and then placed in distilled water without arsenite to complete the incubation period [53,54]. Due to the high sensitivity of *Allium cepa* to sodium arsenite exposure reported in two studies [55,56], it was decided to use an exposure time of only one hour at 0.37 µg/mL, to prevent masking of genotoxic damage by the cytotoxic effects (induction of apoptosis and necrosis). The onions exposed to sodium arsenite were incubated for 71 h extra in distilled water to resemble the conditions used for AgNPs and AgNO_3_. Three independent experiments by triplicate were performed for each treatment.

### 2.3. Sample Preparation

After incubation time, three mm of the root was fixed with MeOH (80% *v*/*v*) and then submerged for 2 min in 5 N HCl. After that, samples were rinsed with distilled water to remove the acid excess. Rinsed roots were submerged in the acetic-orcein stain for 30 min and then rinsed with distilled water. Finally, the stained root was placed on a slide with a drop of acetic acid at 45% (*v*/*v*). The sample was “squashed” with the help of a coverslip for microscope observation. Observations were performed with a Carl Zeiss Primo Star microscope (Carl Zeiss Microscopy GmbH, Jena, Germany) with a 40× objective. 

### 2.4. Mitotic Index and Genotoxicity

The mitotic index was determined with the ratio of cells in division (P = prophase + M = metaphase + A = anaphase + T = telophase) and the total number of counted cells according to the formula:

MI = [(Cells on division (P + M + A + T))/(Total counted cells)] × 100(1)

Genotoxicity was determined with the micronuclei frequency present on 1000 cells under division counted to determine the mitotic index [57]. 

### 2.5. Determination of Antioxidant Capacity

The antioxidant capacity was determined using the Oxygen Radical Activity Capacity kit (ORAC kit, ab233473, Abcam, Cambridge, MA, USA) according to the method described by [58]. Briefly, one gram of freeze-dried extract (H_2_O: MeOH, 20: 80 *v*/*v*) of *Allium cepa* roots and bulbs were diluted in methanol for quantification. Analyses were performed at 37 °C using a pH 7.4 phosphate buffer. The peroxide radicals were produced by 2,2′-Azobis(2-amidinopropane) dihydrochloride (AAPH), using fluorescein as substrate and Trolox as standard. Fluorescence was measured every 2 min for one hour. A calibration curve of Trolox in the concentration range 10 to 100 μM was used in each plate read. All determinations were done by triplicate.

### 2.6. Determination of Reactive Oxygen Species (ROS)

The determination of ROS was performed by a direct colorimetric and fluorometric assay that measures hydrogen peroxide (H_2_O_2_) as a reactive oxygen metabolic by-product (Hydrogen Peroxide Assay Kit-ab102500, Abcam, Cambridge, MA, USA). The determination was performed following the supplier protocol. Briefly, 5mg of freeze-dried *Allium cepa* roots and bulbs samples were separately homogenized in cold phosphate buffer solution and washed by centrifugation for 2–5 min at 4 °C and 1000× *g* to remove any insoluble material. The collected supernatant was transferred to a clean tube to keep on ice. Perchloric acid (PCA) 1 M was used for deproteination; the mixture was stirred and incubated on ice for 5 min. PCA was precipitated with 2M KOH. The mixture was centrifuged at 10,000× *g* for 20 min at 4 °C, and the supernatant was collected. Deproteinized samples were used to determine ROS with Hydrogen Peroxide Assay Kit (Abcam, Cambridge, MA, USA). All determinations were performed by triplicate.

### 2.7. Determination of Total Phenolic Content (TPC)

Samples from roots and bulbs from the different experimental conditions were extracted for three hours at 250 rpm with a solvent mixture H_2_O: MeOH (50:50 *v*/*v*) at 30 °C. The obtained extracts were filtered under vacuum and concentrated in a rotary evaporator. The concentrated extract was lyophilized, and the obtained freeze-dried powder was stored at −80 °C. The TPC was determined using the Folin–Ciocalteu method previously described by [50]. The absorbance was measured at 760 nm, and TPC was calculated from a calibration curve of gallic acid (10–150 μg/mL) and expressed as milligrams of gallic acid equivalents (GAE) per gram of sample. All assays were carried out in triplicate.

### 2.8. Determination of Lipoperoxidation (LPO)

Lipid peroxidation was determined indirectly by the quantification of malondialdehyde (MDA) produced by the decomposition of unsaturated fatty acids. 200 mg of freeze-dried roots and bulbs samples were homogenized in 4 mL of 0.1% Trichloroacetic acid (TCA). The extract was centrifugated at 10,000× *g* for 15 min. 1 mL of supernatant was collected and mixed with 2 mL of 20% TCA and 2 mL of 0.5% Thiobarbituric acid (TBA). The mixture was heated for 30 min at 95 °C, then cooled on ice. The produced malondialdehyde was quantified reading at 532 and 600 nm. All determinations were performed by triplicate. 

### 2.9. Statistical Analysis

GraphPad Prism 8.4 was used to analyze data, which are expressed as the means ± standard error. One-way ANOVA statistical analysis was performed, followed by Tukey’s test to identify significant differences among groups. Significant differences were considered with *p* < 0.05. A Bartlett test [59] was performed before conduct each analysis of variance to probe the null hypothesis that variances in all groups are the same. The results showed *p* ≥ 0.05 for all variables considered in this study. We assume normality based on the Bartlett test sensitivity for normal distributions [60].

## 3. Results and Discussion

**Figure 1:**
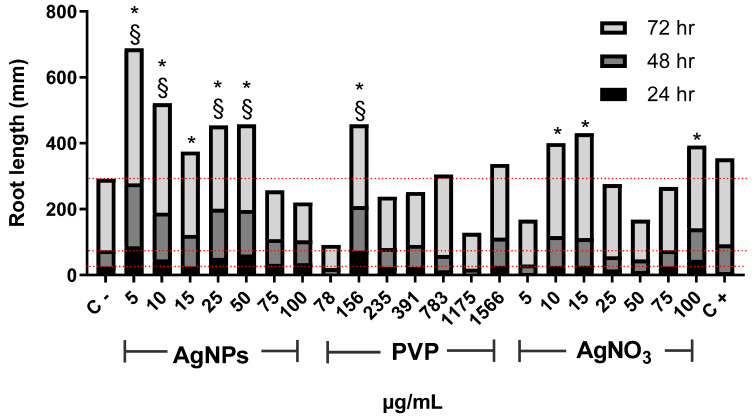
Root elongation of *Allium cepa* exposed to AgNPs, PVP, and AgNO_3_ with different concentrations after 24 (black), 48 (dark gray), and 72 h (gray) of exposure. The negative control (C−) was distilled water, and 0.37 µg/mL of sodium arsenite was used as a positive control (C+). Dotted lines were included for comparative purposes that show the elongation observed for negative control on each evaluated time. * Indicates significative differences with the negative control (*p* < 0.05); § indicates significative differences with the positive control (*p* < 0.05) after 72 h of exposure.

Figure 1 shows changes in *Allium cepa* root length with time. After 24 h of exposure, AgNPs with concentrations of 5, 10, 25, and 50 µg/mL, as well as 156 µg/mL for PVP, promoted root elongation compared with the negative control. For this exposure time, the most important elongation was observed for 5 µg/mL of AgNPs. The lowest concentrations of PVP (78 µg/mL) and AgNO_3_ (5 µg/mL) seem to make root elongation slower. Root elongation increase was observed with the concentration increase of both PVP and AgNO_3_, but to less degree than the obtained for AgNPs. The minimal root elongation was found in onions exposed to 1175 and 50 µg/mL of PVP and AgNO_3_, respectively (Figure 1). Most significant changes in root elongation were observed on plants exposed to AgNPs for 48 h, being the most impressive one reached in onion exposed to 5 µg/mL of AgNPs, 3.5-times higher elongation compared with the negative control (Figure 1). PVP and silver nitrate showed similarly or slightly superior elongation values than the negative control, albeit never more than double. After 72 h of exposure, the highest root elongation was still produced by the lowest concentrations of AgNPs assayed, 5 µg/mL and 10 µg/mL.

**Figure 2:**
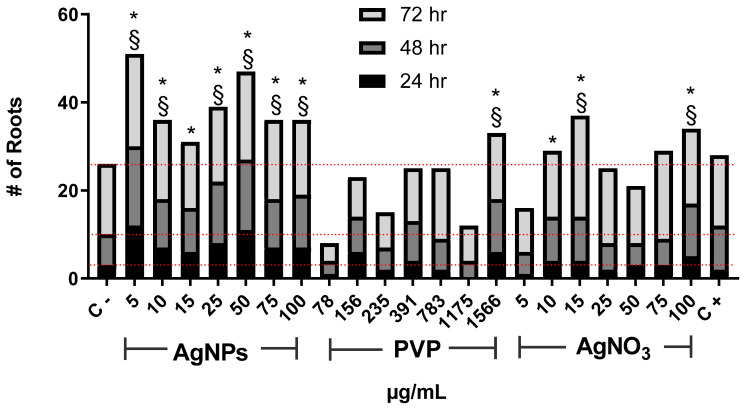
The number of new roots counted on *Allium cepa* exposed to AgNPs, PVP, and AgNO_3_ with different concentrations after 24 (black), 48 (dark gray), and 72 h (gray) of exposure. Negative control (C−) was distilled water. 0.37 µg/mL of sodium arsenite was used as a positive control (C+). Red dotted lines were included for comparative purposes that show the number of roots observed for negative control on each evaluated time. * Indicates significative differences with the negative control (*p* < 0.05); § indicates significative differences with the positive control *(p* < 0.05) after 72 h of exposure.

The number of new roots found after the exposure to AgNPs increases for all assessed concentrations compared with the negative control (Figure 2). As in the case of root elongation, the concentration of 5 µg/mL was the most effective. Interestingly, the number of new roots found for 10, and 15 µg/mL rapidly drops compared with those seen for 5 µg/mL. Then it increases again for concentrations of 25 and 50 µg/mL, but not so impressive as for 5 µg/mL. The number of roots found for 75 and 100 µg/mL drops again.

On the other hand, PVP only promoted the emergence of new roots with the highest concentration assessed, 1566 µg/mL. Meanwhile, AgNO_3_ shows the changing pattern found for different concentrations of AgNPs but, in this case, involving the concentrations from 15 (maximum root numbers) to 100 µg/mL. For both agents, PVP, and AgNO_3_, the lowest concentration assessed presents the smaller number of new roots, even lower than for the negative control (Figure 2).

**Figure 3:**
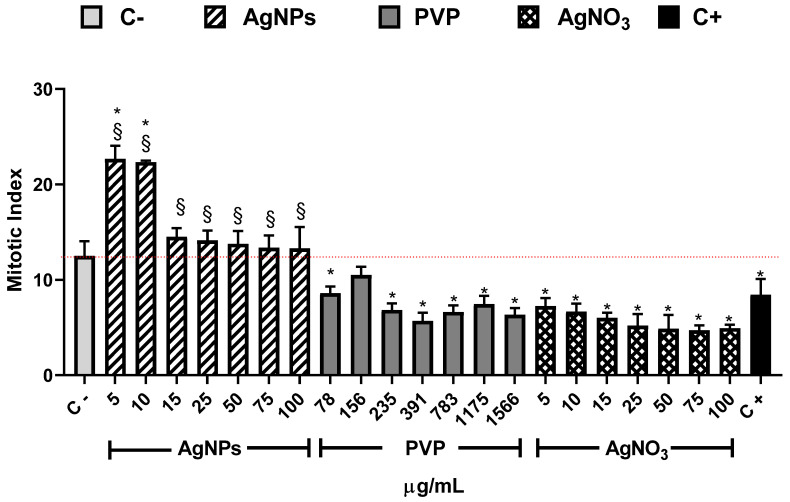
Mitotic index obtained after 72 h of exposure to the corresponding stimuli, AgNPs (lined), PVP (dark gray), and AgNO_3_ (grid). The concentrations assessed are indicated in the figure. C− corresponds to untreated plants (light gray) and C+ to those exposed to 0.37 µg/mL of sodium arsenite (black). * Indicates significative differences with the negative control (*p* < 0.05); § indicates significative differences with the positive control (*p* < 0.05).

The mitotic index shown in Figure 3 is the primary biomarker used to determine the cytotoxic effect of different substances and provides strong arguments to explain the root elongation and increase of root number elicited by exposure of *Allium cepa* to AgNPs. In our experimental conditions, the MI value for the negative control (C−) was 12.5 ± 1.5. This value is similar to the reported by Dizdari [61] and Cvjetko [14] with IM values of 15 ± 0.32 and 9 ± 0.5, respectively. 

**Figure 4:**
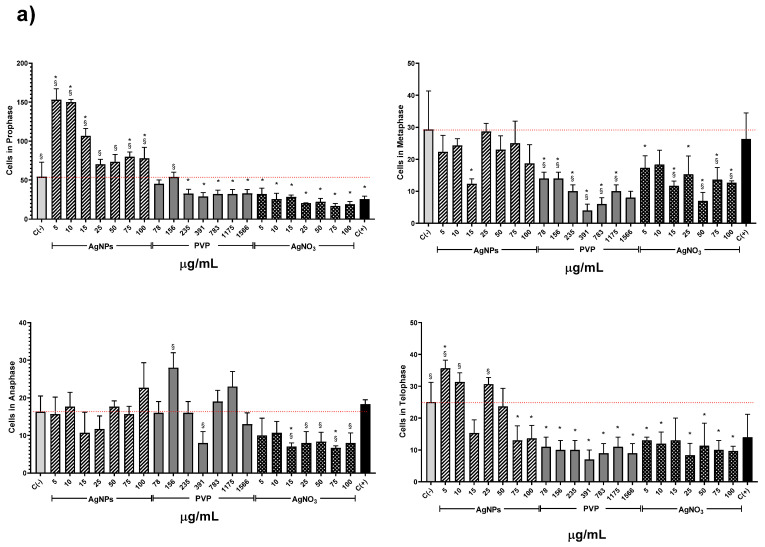

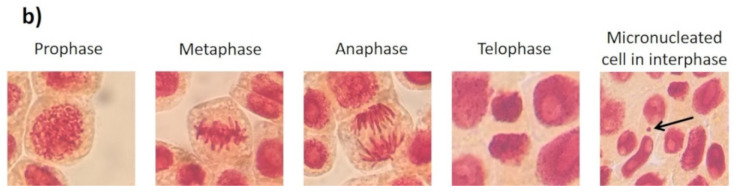
Effects elicited by AgNPs, PVP, and AgNO_3_ on mitosis of *Allium cepa* root cells. (**a**) Cell population in each phase of mitosis after 72 h of exposure to several concentrations of AgNPs (lined), PVP (dark gray), and AgNO_3_ (grid). C- corresponds to untreated plants (light gray) and C+ to those exposed to 0.37 µg/mL of sodium arsenite (black). * Indicates significative differences with the negative control *(p* < 0.05); § indicates significative differences with the positive control (*p* < 0.05). (**b**) Representative photographs of cells at different stages of mitosis. Images were obtained with a digital camera adapted to the microscope using a 40× objective.

The AgNPs concentrations of 5 and 10 µg/mL showed higher MI values for all the series. Meanwhile, the other concentrations (15–00 µg/mL) showed a MI value similar to the negative control, but never below. On the contrary, for all PVP and AgNO_3_ concentrations, the MI values are beneath the negative control. MI value ranges are within 8.6 ± 0.7–6.3 ± 0.7 for PVP and 7.2 ± 0.8–4.7 ± 0.5 for AgNO_3_. MI value for sodium arsenite is close to the MI value of PVP (8.43 ± 1.66). From 5 to 25 µg/mL of silver nitrate, the MI decreases in a concentration-dependent manner; for 50 µg/mL and higher concentrations, the MI keeps practically constant (Figure 3). It is clear from Figure 3 that lower concentrations of this AgNPs formulation promote cellular division, contrary to silver ions that affect cell division starting from the lower concentration assessed. A detailed analysis of cell populations demonstrates that the exposure to AgNPs with concentrations of 5 and 10 µg/mL elicits a critical percentage of cells found in prophase–more than three times in comparison with the negative control (Figure 4). Additionally, a small increase in the frequency of cells in telophase is observed with these concentrations. With higher concentrations of AgNPs (75 and 100 µg/mL), the frequency of cells on prophase is still above the observed for the negative control.

Conversely, PVP and AgNO_3_ decrease the cell count in all phases compared with the negative control, except for the interphase. Both agents present a cell counting decrease on prophase with a dose-concentration behavior. For the rest of the phases, no dose-dependence behavior was found, but in all of them, a significant reduction in cell counting compared with the negative control was observed, even most important than the produced by sodium arsenite (C+).

**Figure 5:**
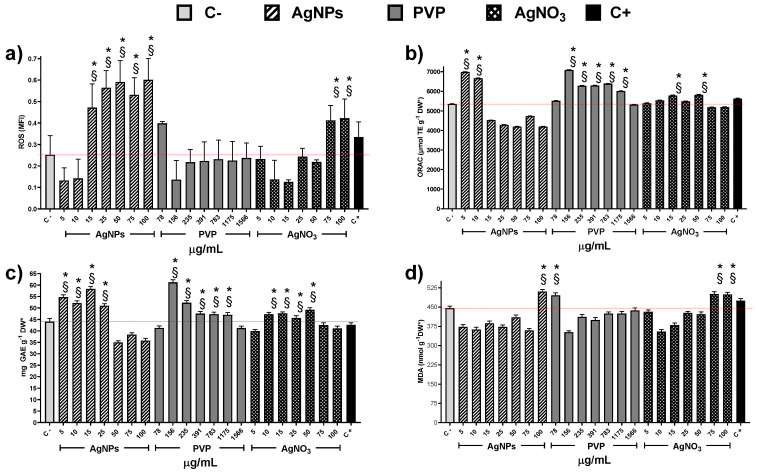
Antioxidant response of *Allium cepa* roots exposed to different stimuli. (**a**) Reactive Oxygen Species, (**b**) Oxygen Radical Absorption Capacity assay, (**c**) Total Phenolic Content, and (**d**) Lipoperoxidation recorded on the onion roots after 72 h for different concentrations of AgNPs (lined), PVP (dark gray), and AgNO_3_ (grid). C- corresponds to untreated plants (light gray) and C+ to those exposed to 0.37 µg/mL of sodium arsenite (black). * Indicates significative differences with the negative control (*p* < 0.05); § indicates significative differences with the positive control (*p* < 0.05).

Exploring the factors that could contribute to cytotoxicity and, in turn, to the decrease of MI values, we quantify the concentration of reactive oxygen species (ROS) within the cells. It is important to note that only PVP at 78 µg/mL and AgNO_3_ at 75 and 100 µg/mL produce an increase of ROS statistically significant compared with the negative control (Figure 5a). On the other hand, AgNPs provide a significate upsurge of ROS starting from the concentration of 15 µg/mL. 

The oxygen radical absorbance capacity registered on plants exposed to AgNPs shows an increase compared with negative control only for concentrations of 5 and 10 µg/mL (Figure 5b), despite the ROS underproduced by these concentrations (Figure 5a). Contrariwise, PVP increases the presence of antioxidant agents for the concentration range of 156 to 1175 µg/mL. Silver ions present practically no changes, except for the concentrations 15 and 50 µg/mL (Figure 5b). Figure 5c shows total phenol content (TPC) as a part of the antioxidant response of the onions to the application of the chemical agents. The TPC uprate for AgNPs was observed in the concentration range 5–25 µg/mL, while for PVP and silver ions in a broader range, 156–1175 and 10–75 µg/mL, respectively. The lipoperoxidation (Figure 5d) only show differences in comparison with the negative control for the high concentrations of AgNPs (100 µg/mL) and Ag^+^ (75 and 100 µg/mL). 

Indeed, these results suggest different cytotoxic mechanisms exerted by the substances evaluated in this work. The MI drop registered in onions exposed to Ag^+^ or PVP did not show a direct association with the overproduction of ROS. On the other hand, the ROS overproduction elicited by AgNPs does not produce changes on the mitotic index compared with the negative control (Figure 3 and Figure 5a). 

Essential differences in the antioxidant response of the plant support the proposal of different cytotoxic mechanisms exerted by these agents. The low concentrations of AgNPs cause an upper production of TPC that helps the enzymatic response to fight ROS overproduction. Meanwhile, at 50 to 100 µg/mL, the TPC decreases 20% compared with the negative control, suggesting that from here on, the antioxidant activity ultimately falls on the enzymatic systems. Nevertheless, even at higher AgNPs concentrations, the onion antioxidant response is still useful because the mitotic index presents no changes, and the frequency of cells on prophase and telophase increases compared with the negative control.

Only the higher concentration of AgNPs evaluated, 100 µg/mL, produces an increase of malondialdehyde that can be considered as the beginning of lipoperoxidation compared with the negative control. So, for low concentrations of AgNPs, no ROS overproduction was observed, but an increase in the antioxidant response was found (increase in ORAC and TPC compared with C-), while for Ag^+^ no changes neither in ROS concentration nor in the antioxidant response was observed. These could explain the drastic root growth activation caused by AgNPs compared with Ag^+^. For high concentrations of AgNPs and Ag^+^, two biomarkers associated with phytotoxicity increase compared with C-: ROS increases by 200% and 120%, respectively, and lipoperoxidation increase 12–14% (Figure 4). Nevertheless, antioxidant mechanisms, measured by ORAC and TPC, show a small decrease with AgNPs and has not been modified for Ag^+^, supporting the hypothesis of different cytotoxic mechanism exerted. 

The results obtained with the onion agree with the hormetic effect produced by the same AgNPs formulation on sugar cane [62], vanilla [50], and stevia [63] through ROS overproduction. Besides, this is important to detect the concentration where growth promotion without adverse effects is observed in onions, and the differences in the antioxidant response compared with other plants already exposed to this type of AgNPs. For onions, cytotoxic damage apparently begins with 100 µg/mL of AgNPs because only a small increase of malondialdehyde is observed (Figure 5c). While on the other plants, with this concentration, the damage is quite evident not only at the molecular level but also physiologically, due to different antioxidant response of these plants [50,62,63].

The ROS overproduction and the antioxidant response on the onion bulb are quite similar to those observed on the roots. The main difference consists of TPC production. In the case of the bulb, silver ions enlarge a little bit TPC with the concentration range 5–50 µg/mL. Meanwhile, PVP does it with the range 156–1175 µg/mL, being the latter one of the most significant TPC values registered here. Therefore, no damage was registered on the bulb with any of the AgNPs concentrations evaluated, considering that MDA registered with exposure to 100 µg/mL of AgNPs is just the beginning of cell damage (Figure S1). 

Thus, at low AgNPs concentrations factors increasing plant growth (oxygen radical absorption capacity, Figure 5b, and total phenolic content, Figure 5c) are maximum with no evidence of cellular damage. The antioxidant response could explain the increase in the number and length of roots and the mitotic index (Figure 1, Figure 2 and Figure 3). 

**Figure 6:**
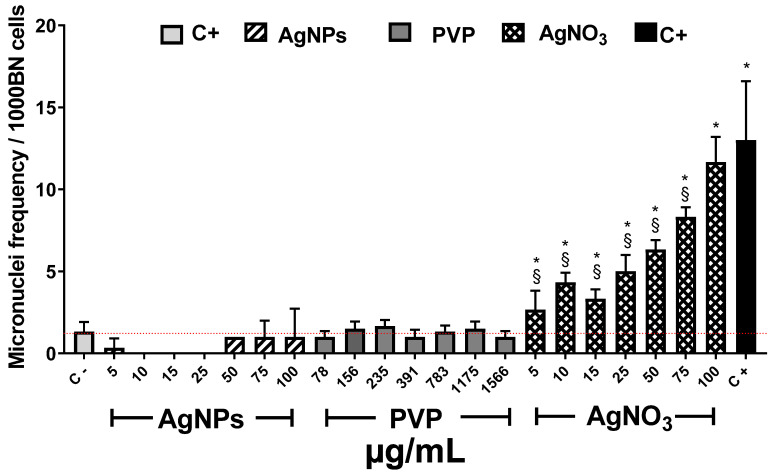
Micronuclei frequency (MN) on *Allium cepa* root exposed to different stimuli. The MNi frequency was recorded after 72 h exposure to different concentrations of AgNPs (lined), PVP (dark gray), and AgNO_3_ (grid). C− corresponds to untreated plants (light gray) and C+ to those exposed to 0.37 µg/mL of sodium arsenite (black). * Indicates significative differences with the negative control (*p* < 0.05); ^§^ indicates significative differences with the positive control (*p* < 0.05).

It is known that one of the consequences of ROS overproduction is reversible or irreversible nuclear material damage [64]. In order to complete the phytotoxic influence of these compounds on *Allium cepa*, the AgNPs genotoxic potency was determined though the recording of micronuclei (MN) frequency. Figure 6 shows the MN frequency observed after 72 h of exposure to the different agents. *Allium cepa* is one of the most sensitive systems for genetic damage assessment. Moreover, the number of chromosomes provides an essential advantage for tracking genetic damage due to the reduced number of chromosomes [25]. 

Several authors reported that MN frequency on basal conditions for *Allium cepa* is between 1 and 2 [65,66,67]. In our experimental conditions, MN counting (1.3 ± 0.5) agrees with those values previously reported. 

As expected, the known genotoxic agent sodium arsenite, exhibited the most significant MN frequency, ten-times higher (13 ± 3.6 MN) than the observed for negative control (1.3 ± 0.5 MN). Contrariwise, exposure to AgNPs at any of the concentrations assayed showed lower values than the recorded for the negative control. Interestingly, no increase in MN frequency was recorded on the samples exposed to AgNPs neither with the low (5 and 10 µg/mL) nor the higher concentration (25–100 µg/mL), despite the latter elicit the highest ROS overproduction (Figure 5b). All assessed PVP concentrations show low MN frequency similar to AgNPs and the negative control. (Figure 6). Contrastively, silver ions duplicate MN frequency (2.6 ± 1.1 MN) compared with negative control (1.3 ± 0.5) starting from the lowest concentration (5 µg/mL). For 100 µg/mL of AgNO_3_, MN frequency reached 11.6 ± 1.5, response quite similar to sodium arsenite (13 ± 3.6 MN). The MN frequency increases with Ag^+^ concentration demonstrating that silver ions display a concentration-dependent behavior. 

It has been reported that low concentrations of silver ions can unidirectionally affect the K^+^ flux decreasing its intracellular concentration, while higher concentrations produce the same effect but damaging cellular membrane [68]. Additionally, silver ions can block the recognition sites of ethylene, avoiding the completeness of the signaling route [69]. The above could explain the decrease in MI and the diminish of cells in prophase and telophase observed in roots exposed to silver ions.

The results obtained in this work with Ag^+^ ions agree with the concentration-dependent phytotoxic effects described by Panda [37] and Yekeen [32] in *Allium cepa*. Panda found a significant decrease in the mitotic index and a substantial increase in the frequency of cells with MN with low concentrations of Ag^+^ ions (5 µg/mL) and only 2 h of exposure [37]. The literature data and the different responses from Allium cepa root cells exposed to silver ions and AgNPs support our proposal of different mechanisms of actions elicited by both agents. 

On the other hand, the cytotoxic and genotoxic response of *Allium cepa* roots after exposure to sodium arsenite show concentration- and time-dependence behavior. It was demonstrated that micronuclei frequency and mitotic index are directly dependent on sodium arsenite exposure time. Both parameters show an opposite trend with prolonged exposure, that is, as longer the exposure time, lower the mitotic index, and higher the micronuclei frequency recorded [55,56]. Sodium arsenite concentrations of 0.3 to 1 μg/mL after 1h of exposure produce a significant statistical difference in the micronuclei frequency with lower affectation in the mitotic index compared with negative control [55,56]. These results show the tremendous cytotoxic and genotoxic damage produced by low concentrations and short exposure times of sodium arsenite in *Allium cepa* root cells. 

In our experimental conditions (0.37 µg/mL and 1 h of exposure), a similar trend than that previously described for sodium arsenite was observed. The length of the roots and appearance of new ones after 24 h (Figure 1 and Figure 2) is lower compared with the negative control, which is consistent with the rapid cytotoxic damage previously described. Moreover, after 72 h, the cytotoxic and genotoxic damage on the root cells exposed to this low concentration of arsenite for a very short time is still measurable, showing a decrease on the mitotic index (Figure 3), a significant reduction of cells in prophase (Figure 4) and a meaningful increase in the micronuclei frequency (Figure 6). All of this is without a considerable difference elicited by arsenite in the antioxidant response, ROS overproduction, total phenol content, or evidence of lipoperoxidation compared with the negative control after 72 h (Figure 5). It is very important to bear in mind that the damage caused by the arsenite must have occurred during the first hours of exposure, but it was so great that even after 72 h of exposure, it is still measurable in parameters such as mitotic index and micronucleus frequency.

On the other hand, low concentrations of AgNPs (5 and 10 µg/mL) produce a rise in the root length promote the appearance of new ones (Figure 1 and Figure 2), increase in the mitotic index (Figure 3) and cells in prophase (Figure 4). These concentrations do not lead to ROS overexpression but increase the total phenol content and the antioxidant response, suggesting that plants grow in order to cut down the possible damage. As the AgNPs concentration increase, noticeable increase the ROS overproduction and the total phenolic content and the antioxidant response decrease. Nevertheless, no differences were observed in the number and length of roots, mitotic index, or the micronuclei frequency compared with the negative control.

These results suggest that *Allium cepa* root cells are better able to handle the possible damage caused by higher concentrations of AgNPs after longer exposure times than the damages caused by a 13 to 270 times lower concentration of arsenite with 72 times less of exposure time than ones applied for AgNPs. In the employed experimental conditions, the damage produced by AgNPs is meager considering the significant damage generated by a low concentration of sodium arsenite after the very short exposure time. However, further experiments must be performed to confirm the lack of cytotoxic and genotoxic damage of the AgNPs formulation evaluated in this work.

All PVP-AgNPs formulations listed in Table 1 produce chromatic aberrations. PVP-AgNPs formulation studied by Cvjetko at a concentration of 10 µg/mL of metallic silver (100 µM) produces DNA damage evidenced by the increase of the comet tail [14]. This concentration, 10 µg/mL, represents only one-tenth of the maximum concentration evaluated for Argovit™ in this work, but the latter did not produce cytotoxic or genotoxic damage even when 100 µg/mL of metallic silver was used. Other PVP-AgNPs with sizes 5, 25, 50, and 75 nm were studied by Scherer at concentrations of 100 µg/mL of the complete nanoparticle formulation observing that the smaller the AgNPs diameter, the more the MI decrease and the MN frequency increases compared to the control group. The concentration of 100 µg/mL of the complete nanoparticle formulation represents the sixteenth part of the Argovit™ concentration used in this work. For Argovit™, 83–666 µg/mL of the complete AgNPs formulation corresponds to 5–100 µg/mL considering the content of metallic silver.

Until this work, all the AgNPs formulations evaluated had shown phytotoxicity on *Allium cepa*. Results obtained in this work show that cytotoxic and genotoxic responses of Argovit™ PVP-AgNPs are less than the effect produced by AgNPs formulations listed on Table 1. The shape, size, and coating agent of the nanoparticles from Table 1 and the evaluated in this work are quite similar, but the latter did not generate phytotoxic damage. The Ag/coating agent ratio is the only factor that could explain the main differences in the toxicological response observed in this work with those previously reported since there are no such dramatic toxicological differences associated with the difference in size, shape or silver content [21,22,38,39,40,41,70,71]. Considering dried nanoparticles, the [Ag]/[PVP] ratio expressed in % of weight in the formulation studied here is 6:94. Meanwhile, NanoComposix is 34:66, and the synthesized by Cvjetko is 40:60 [20]. Unfortunately, we have not found information about Nanotech Ltd.’s formulation. 

Hence, even though the concentration of AgNPs studied in this work was at least 10–17 times higher than those for previously reported PVP-AgNPs formulations, no cytotoxic nor genotoxic damage for *Allium cepa* was observed. Lack of damage under the experimental conditions assessed could be a good sign regarding their environmental impact, but further experiments with more extended exposure periods must be performed to determine chronic toxicity effects.

## 4. Conclusions

In this work, the cytotoxic and genotoxic effect of AgNPs formulation Argovit™ for *Allium cepa* (onion), a recognized reference system for higher plants, were studied. Our results allow us to conclude that this AgNPs formulation produces no cytotoxic nor genotoxic damage at the concentrations assessed on *Allium cepa* compared with other PVP-AgNPs formulations reported on literature. Comparative analysis of the behavior of Argovit™ AgNPs and AgNO_3_ showed that the primary biological effect of Argovit™ is not associated with the released silver ions but to AgNPs themselves. Furthermore, our results show the relevance of evaluating the cyto-genotoxic response of the coating agent since the PVP considered as non-toxic and, therefore, frequently used, caused a significant decrease in the mitotic index of onions exposed to this agent. 

The concentrations used in this work for Argovit™ (5–100 µg/mL of metallic silver content or 83–1666 µg/mL of the complete formulation) are 10–17 times higher than the previously reported. It was suggested that the lack of damage elicited by Argovit™ is due to the high proportion of PVP used during their synthesis. A large amount of coating agent could provide to this formulation higher stability and a completely different biological response compared either with other PVP-AgNPs formulations previously reported or to the silver ions. 

In the employed experimental conditions and considering the significant damage generated by a low concentration of sodium arsenite after a very short exposure time, the damage produced by AgNPs is meager. This response could be useful for many applications, particularly low concentrations of Argovit™ that stimulate the growth of onions with minimal cytotoxic or genotoxic damage to the roots or the bulb, also increasing the total phenolic content. 

Results obtained in this work provide valuable information regarding safer nanomaterials design for therapeutic, biomedical, agrochemical, food, and daily use products by modifying the metal/coating agent ratio. These results will be beneficial for widely used nanomaterials design, such as silver nanoparticles and many other nanoparticles whose production begins to increase nowadays due to their full applications.

## Supplementary Materials

The following are available online at https://www.mdpi.com/2079-4991/10/7/1386/s1, Figure S1. Antioxidant response of *Allium cepa* bulbs exposed to different stimuli.
