# Supplementary material for: Argovit™ Silver Nanoparticles Effects on Allium cepa: Plant Growth Promotion without Cyto Genotoxic Damage
Source: Nanomaterials (Basel). 2020 Jul 16;10(7):1386. doi: 10.3390/nano10071386 (PMC7408422; doi:10.3390/nano10071386)
Supplement: Supplementary file 1 [file nanomaterials-10-01386-s001.pdf]

nanomaterials

Supplementary Materials

Argovit™ Silver Nanoparticles Effects on *Allium cepa*: Plant Growth Promotion without Cyto-Genotoxic Damage

Francisco Casillas-Figueroa [1], María Evarista Arellano-García [2,*], Claudia Leyva-Aguilera [2], Balam Ruíz-Ruíz [3], Roberto Luna Vázquez-Gómez [1], Patricia Radilla-Chávez [1], Rocío Alejandra Chávez-Santoscoy [4], Alexey Pestryakov [5], Yanis Toledano-Magaña [1], Juan Carlos García-Ramos [1,*] and Nina Bogdanchikova [6]

[1] Escuela de Ciencias de la Salud, UABC, Blvd. Zertuche y Blvd., De los Lagos S/N Fracc, Valle Dorado, 22890 Ensenada, Baja California, Mexico; casillas.francisco@uabc.edu.mx (F.C.-F.); cuentarluna@gmail.com (R.L.V.-G.); patyradilla@uabc.edu.mx (P.R.-C.); yanis.toledano@uabc.edu.mx (Y.T.-M.)
[2] Facultad de Ciencias, UABC, Carretera Transpeninsular Ensenada-Tijuana No. 3917 Col. Playitas, 22860 Ensenada, Baja California, Mexico; cleyva@uabc.edu.mx
[3] Facultad de Medicina extensión los Mochis, Universidad Autónoma de Sinaloa, Av. Ángel Flores s/n, Ciudad Universitaria, 81223 Los Mochis, Sinaloa, Mexico; balamruiz@gmail.com
[4] Centro de Biotecnología-FEMSA, Escuela de Ingeniería y Ciencias, ITESM,Monterrey, Eugenio Garza Sada, 2501 Sur, 64849 Monterrey, Nuevo León, Mexico; chavez.santoscoy@tec.mx
[5] Department of Technology of Organic Substances and Polymer Materials, Tomsk Polytechnic University, 634050 Tomsk, Russia; pestryakov2005@yandex.ru
[6] Centro de Nanociencias y Nanotecnología, UNAM, Carretera Tijuana-Ensenada Km 107, 22860 Ensenada, Baja California, Mexico; nina@cnyn.unam.mx
* Correspondence: evarista.arellano@uabc.edu.mx (M.E.A.-G.); juan.carlos.garcia.ramos@uabc.edu.mx (J.C.G.-R.); Tel.: +52-64-6174-5925 (ext. 225) (M.E.A.-G.); +52-64-6175-0707 (ext. 65313) (J.C.G.-R.)

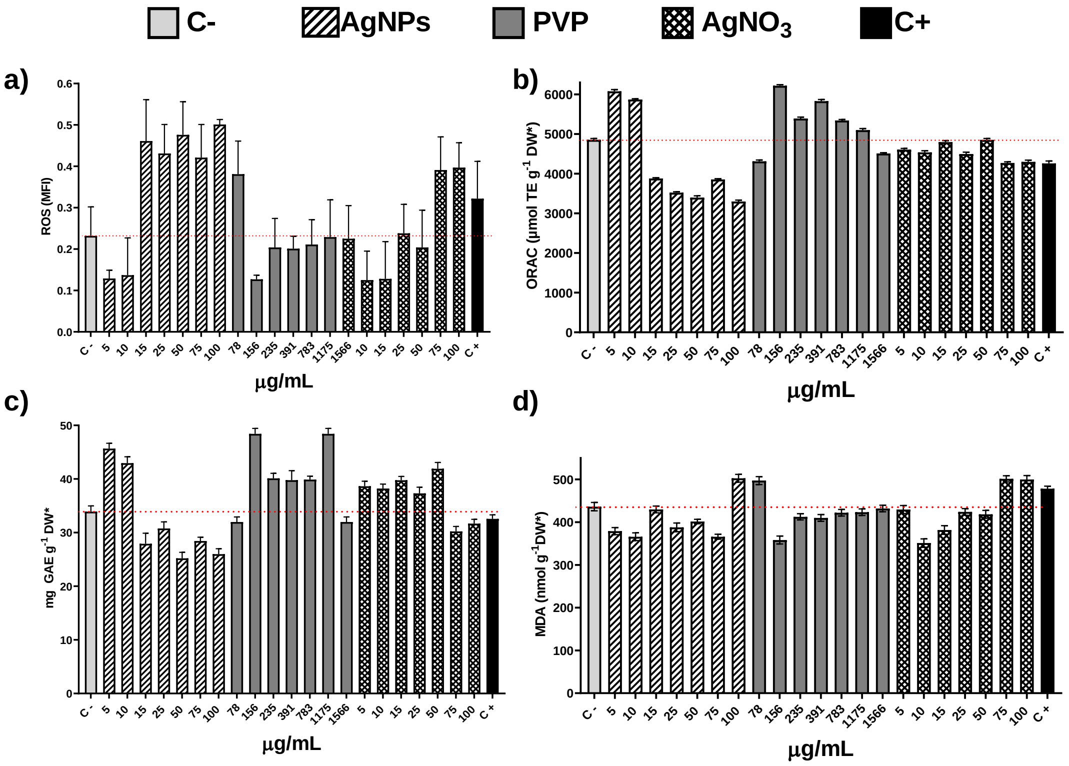

Figure S1. Antioxidant response of *Allium cepa* bulbs exposed to different stimulus. (**a**) Reactive Oxygen Species, (**b**) Oxygen Radical Absorption Capacity assay, (**c**) Total Phenolic Content, and (**d**) Lipoperoxidation recorded on the onion roots after 72h for different concentrations of AgNPs (lined), PVP (dark gray), and $AgNO_3$ (grid). C− corresponds to untreated plants (light gray) and C+ to those exposed to 0.37 µg/mL of sodium arsenite (black). * indicates significative differences with the negative control ($p < 0.05$); § indicates significative differences with the positive control ($p < 0.05$).
